# Entorhinal and Transentorhinal Atrophy in Preclinical Alzheimer's Disease

**DOI:** 10.3389/fnins.2020.00804

**Published:** 2020-08-21

**Authors:** Sue Kulason, Eileen Xu, Daniel J. Tward, Arnold Bakker, Marilyn Albert, Laurent Younes, Michael I. Miller

**Affiliations:** ^1^Center for Imaging Science, Johns Hopkins University, Baltimore, MD, United States; ^2^Institute for Computational Medicine, Johns Hopkins University, Baltimore, MD, United States; ^3^Department of Biomedical Engineering, Johns Hopkins University, Baltimore, MD, United States; ^4^Department of Computational Medicine, University of California, Los Angeles, Los Angeles, CA, United States; ^5^Department of Neurology, Ahmanson-Lovelace Brain Mapping Center, University of California, Los Angeles, Los Angeles, CA, United States; ^6^Department of Psychiatry and Behavioral Sciences, Johns Hopkins University, Baltimore, MD, United States; ^7^Department of Neurology, Johns Hopkins University, Baltimore, MD, United States; ^8^Department of Applied Mathematics and Statistics, Johns Hopkins University, Baltimore, MD, United States; ^9^Kavli Neuroscience Discovery Institute, Johns Hopkins University, Baltimore, MD, United States

**Keywords:** transentorhinal, entorhinal, preclinical, cortical thickness, change point, diffeomorphometry

## Abstract

This study examines the atrophy patterns in the entorhinal and transentorhinal cortices of subjects that converted from normal cognition to mild cognitive impairment. The regions were manually segmented from 3T MRI, then corrected for variability in boundary definition over time using an automated approach called longitudinal diffeomorphometry. Cortical thickness was calculated by deforming the gray matter-white matter boundary surface to the pial surface using an approach called normal geodesic flow. The surface was parcellated based on four atlases using large deformation diffeomorphic metric mapping. Average cortical thickness was calculated for (1) manually-defined entorhinal cortex, and (2) manually-defined transentorhinal cortex. Group-wise difference analysis was applied to determine where atrophy occurred, and change point analysis was applied to determine when atrophy started to occur. The results showed that by the time a diagnosis of mild cognitive impairment is made, the transentorhinal cortex and entorhinal cortex was up to 0.6 mm thinner than a control with normal cognition. A change point in atrophy rate was detected in the transentorhinal cortex 9–14 years prior to a diagnosis of mild cognitive impairment, and in the entorhinal cortex 8–11 years prior. The findings are consistent with autopsy findings that demonstrate neuronal changes in the transentorhinal cortex before the entorhinal cortex.

## 1. Background

Evidence suggests that neuropathological changes of Alzheimer's disease (AD) begin years before the onset of clinical symptoms (Sperling et al., 2011). Accumulation of these neuropathological changes is associated with neuronal injury, which can be measured indirectly by structural magnetic resonance imaging (MRI) (Atiya et al., 2003; Kantarci and Jack, 2004). A number of MRI studies have detected atrophy in the entorhinal cortex (ERC), hippocampus and amygdala associated with clinical disease severity (Devanand et al., 2007; La Joie et al., 2012; Miller et al., 2015b) and years to AD dementia conversion (Atiya et al., 2003; Kantarci and Jack, 2003). More recent MRI studies have focused on evidence of atrophy that precede clinical symptoms (Jack et al., 2004; Csernansky et al., 2005; den Heijer et al., 2006; Apostolova et al., 2010; Dickerson et al., 2011; Miller et al., 2013; Soldan et al., 2015; Pettigrew et al., 2016), often detecting these smaller changes using time-series data analysis (Durrleman et al., 2012) and survival analysis. These MRI biomarkers of AD-related atrophy prior to manifestation of clinical symptoms are of interest because (1) they may aid in assessing efficacy of therapeutic interventions and (2) they may aid in the identification of populations that can benefit from therapeutic intervention prior to clinical symptoms.

Braak's staging of AD suggests that cortical accumulation of neurofibrillary tangles starts in the transentorhinal cortex (TEC), spreads medially to the ERC, and then involves the hippocampus and amygdala (Braak and Braak, 1991; Braak et al., 2006). Accumulation of tau pathology has been correlated with changes in cognitive status in the presence of β-amyloid plaques (Nelson et al., 2012), as well as with TEC atrophy as detected in MRI (Xie et al., 2018). While relatively few MRI studies exist on AD-related TEC changes (Tward et al., 2017b; Wolk et al., 2017), our recent work demonstrated that subjects with mild cognitive impairment (MCI) have increased baseline atrophy and increased rate of atrophy compared to cognitively normal controls, and that changes in the TEC had greater magnitude than changes in the ERC, hippocampus, and amygdala (Kulason et al., 2019). In addition, our group's work on change point analysis has demonstrated MRI-based shape metrics can detect atrophy in the ERC earlier than hippocampal and amygdalar atrophy (Younes et al., 2014). These changes precede clinical symptoms by up to 10 years.

The evidence of MRI biomarkers that precede clinical symptoms taken together with evidence of cortical AD-related changes selectively occurring first in the TEC motivate a close look at TEC atrophy over the preclinical stage of AD progression. In this study, we aim to localize, both spatially and temporally, MRI-based atrophy detection within the TEC and ERC by examining subjects from two diagnostic groups: stable normal cognition (NC), and NC to MCI converters.

## 2. Methods

### 2.1. Data Collection

Subjects were selected from the ADNI database (adni.loni.usc.edu). The criteria for stable NC included the absence of a diagnosis of MCI or AD on all baseline and follow-up visits, a CDR score of 0 on all baseline and follow-up visits, evidence of performance within the normal range on the Logical Memory Subtest of the Wechsler Memory Scale on all baseline and follow-up visits (based on education adjusted norms), and negative results for elevated amyloid β levels on the baseline visit (greater than a cut off of 192 pg/mL from CSF as established by the ADNI Biospecimen Core).

The criteria for NC to MCI converters included evidence of performance within the normal range on the Logical Memory Subtest of the Wechsler Memory Scale at baseline (based on education adjusted norms), a CDR score of 0 on the baseline exam, a diagnosis of NC at baseline, and a diagnosis of MCI or dementia at a subsequent follow-up visit. Estimated MCI age-of-onset was established based on annual assessment of diagnosis. Note that subjects missing a diagnostic evaluation more than a year prior to MCI diagnosis were excluded, and one subject with an MCI diagnosis was also excluded due to a stable, high score on the Logical Memory Subtest of the Weschler Memory Scale 5 years after diagnosis.

In addition, subjects had to have a minimum of three 3T MRI scans over 2 or more years. Out of the 30 subjects that met all criteria for NC to MCI converters, all subjects were examined and 17 had a continuous collateral sulcus (CoS) and were included in this study (see section 2.2 for detailed explanation). We examined a subset of available stable NC subjects to reach a total sample size of 50. Out of the 84 subjects that met all criteria for stable NC, 68 were examined and 33 had a continuous collateral sulcus and were included in this study. The demographics of subjects included in this study are summarized in Table 1. Two-sample t-tests showed no significant diagnostic group differences by age, number of scans, scan period, or clinical evaluation period. Pearson chi-squared test showed no significant diagnostic group difference by sex. Note that the clinical evaluation period is longer than the scan period because the scan protocol was updated in the ADNI 3 cohort to an accelerated scan sequence. These accelerated scans were not included in this analysis.

**Table 1:** Demographics (mean ± standard deviation where applicable).

**Diagnostic group**	**Stable NC**	**NC to MCI**
Sample size (n)	33	17
Baseline age (years)	72.3 ± 5.5	74.9 ± 5.3
Sex (% Female)	45.5	70.6
# of scans (years)	4.5 ± 0.6	4.6 ± 1.1
Scan period (years)	3.4 ± 1.1	2.9 ± 1.0
Clinical evaluation period (years)	5.3 ± 2.4	6.4 ± 3.7

### 2.2. Manual Segmentation and Surface-Based Morphometry

**Figure 1:**
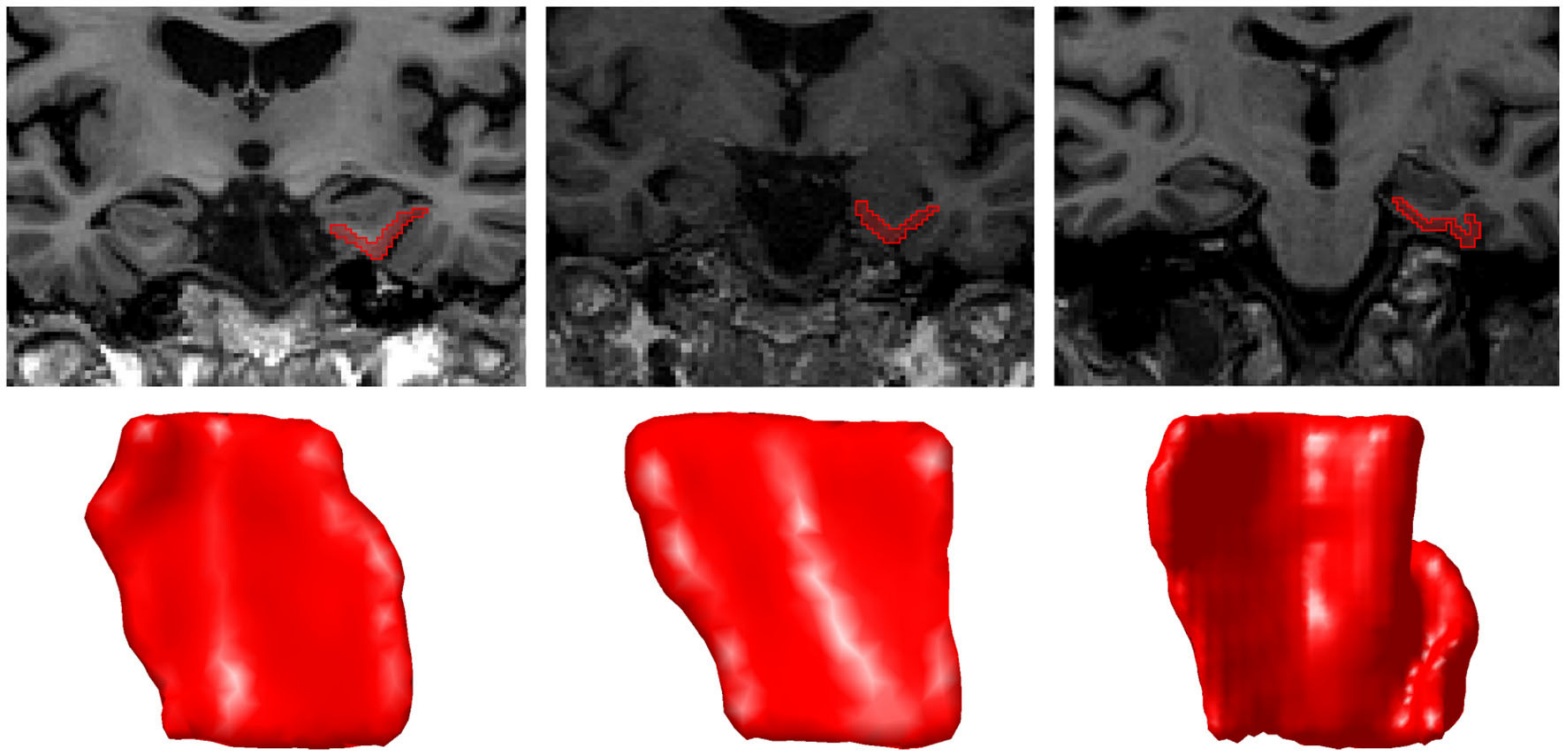
A coronal MRI section and corresponding surface of each CoS variant Type I **(left)**, Type IIa **(middle)**, and Type IIb **(right)**. In Type IIb, the segmentation was extended to the second CoS, also known as the collateral sulcus proper. The orientation of surfaces is as follows: anterior (top), posterior (bottom), medial (left), lateral (right).

As in previous projects (Tward et al., 2017b; Kulason et al., 2019), we restricted the analysis to the left hemisphere and excluded subjects with a discontinuous CoS in the defined region of interest. There are several variants of the CoS to consider, illustrated in Figure 1. The first variant is a deep, continuous sulcus where the rhinal sulcus shares a sulcal bed with the collateral sulcus proper. This variant has been referred to as Type I CoS (Ding and Van Hoesen, 2010) and Type II/Type III rhinal sulcus (Huntgeburth and Petrides, 2012). The second variant is a discontinuous CoS where the collateral sulcus proper begins posterior to the GI. This variant has been referred to as a Type IIa CoS (Ding and Van Hoesen, 2010) and Type I rhinal sulcus (Huntgeburth and Petrides, 2012). Finally, there is a variant with a discontinuous CoS where the collateral sulcus proper begins anterior to the GI, and therefore is excluded for this study. This variant has been referred to as a Type IIb CoS (Ding and Van Hoesen, 2010) and also falls into the category for Type I rhinal sulcus (Huntgeburth and Petrides, 2012). Table 2 categorizes the proportion of CoS variants prior to exclusion of Type IIb. Previous work has shown there is a relationship between CoS depth and the boundaries of the ERC and TEC with respect to anatomical markers (Insausti et al., 1998). Briefly, in a shallow CoS (< 1 cm) the ERC extends to the deepest extent of the CoS. In a regular CoS (between 1 and 1.5 cm), the ERC extends to the midpoint of the medial bank of the CoS. In a deep CoS (> 1.5 cm) the ERC extends up to the CoS. In this subject set we found that excluded Type IIb CoS subjects most often had a shallow CoS, while the Type I and Type IIa variants included in this study were of a regular to deep CoS type.

**Table 2:** Distribution of collateral sulcus variants.

	**Type I (continuous CoS)**	**Type IIa (discontinuous posterior CoS)**	**Type IIb (discontinuous anterior CoS)**
NC (*n* = 68)	21 (31%)	12 (18%)	35 (51%)
NC to MCI (*n* = 30)	13 (43%)	4 (13%)	13 (43%)
Total (*n* = 98)	34 (35%)	16 (16%)	48 (49%)

*Type IIb CoS were excluded from this analysis*.

226 3T T1 MRI scans were used in this study. ERC and TEC were segmented manually using Seg3D software (Center for Integrative Biomedical Computing, 2016). We followed an established procedure for segmentation and delineation of the ERC and TEC (Tward et al., 2017b) that was based on anatomical landmarks described near cytoarchitectonically-defined ERC boundaries (Insausti et al., 1998; Ding and Van Hoesen, 2010). The anterior boundary of the ERC and TEC were defined 4 mm anterior to the hippocampal head. Delineation of the ERC and TEC anterior to this boundary is more complex and excluded from this study. Earlier works suggest that the area anterior to this region is a mix of ERC and perirhinal cortex (Insausti et al., 1998), or olfactory cortex (Krimer et al., 1997), whereas a more recent work suggests that this area is, in fact, part of the ERC (Ding and Van Hoesen, 2010). The posterior boundary for ERC and TEC was defined 2 mm posterior to the gyrus intralimbicus (GI) (Insausti et al., 1998). The medial extent of the ERC was defined as far as the gray/white boundary was visible. This delineation excludes a small dorsal medial aspect of the ERC that rests against the amygdala, and is similar to how other T1 MRI protocols delineate the ERC (Desikan et al., 2006; Maass et al., 2015). The lateral extent of the ERC and medial extent of the TEC was defined at the medial extent of the collateral sulcus (CoS), as is found in a deep CoS (Insausti et al., 1998). The lateral extent of the TEC was defined as being at the deepest extent of the CoS, as is also found in a deep CoS (Ding and Van Hoesen, 2010).

**Figure 2:**
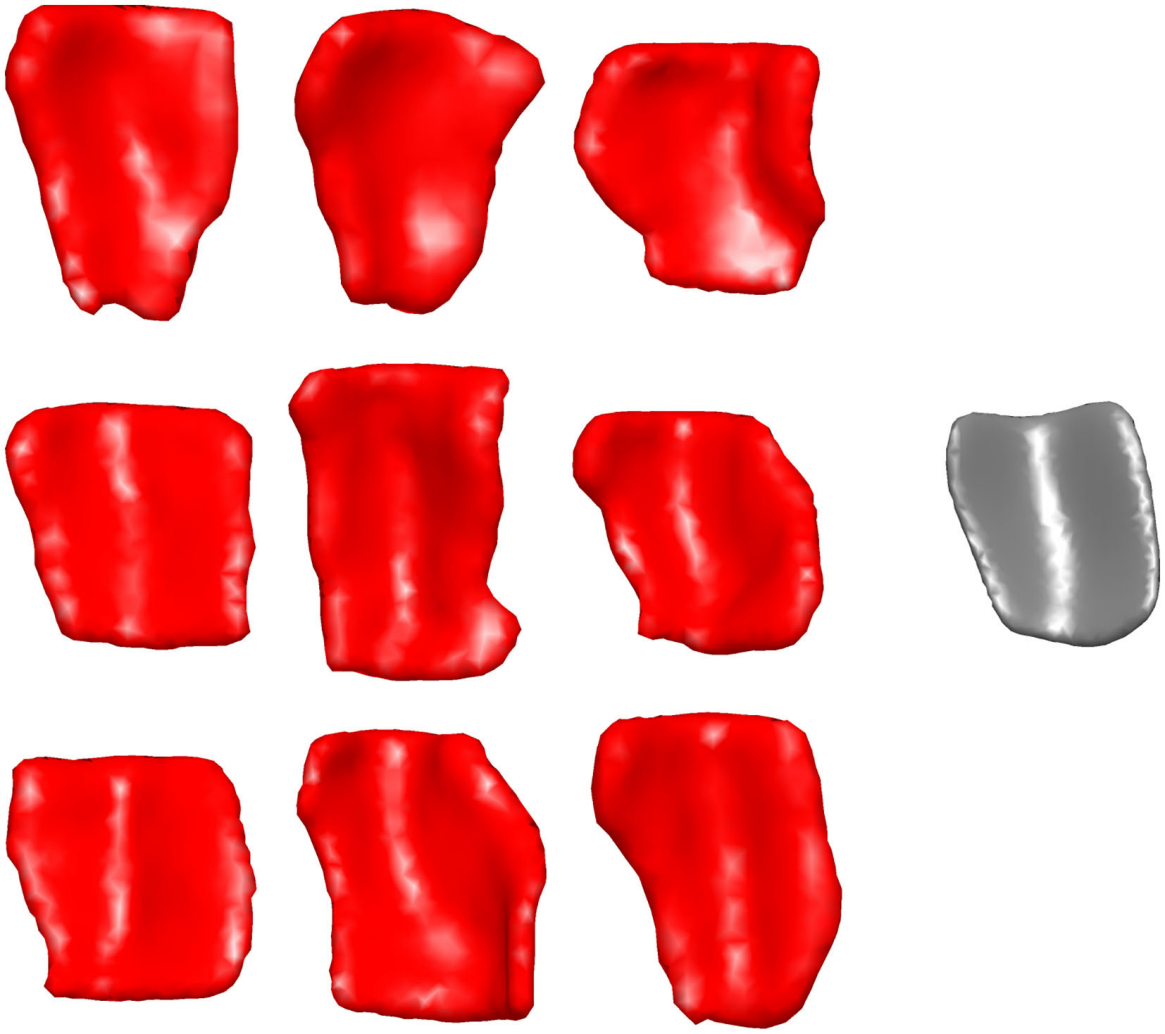
Sample of ERC plus TEC surfaces generated from manual segmentations **(left)** and the resultant population template **(right)**. The orientation is as follows: anterior (top), posterior (bottom), medial (left), lateral (right).

Figure 2 shows a sample of ERC plus TEC surfaces generated from manual segmentations. A population template for the ERC plus TEC surface was calculated from these surfaces by taking the average (Fréchet mean) diffeomorphism in a Bayesian setting (Ma et al., 2008). Then, the segmentations were adjusted for variability in boundaries over time by mapping the population template simultaneously onto each scan of a time series (Tward et al., 2017a). The result is a set of surfaces of the ERC plus TEC.

### 2.3. Surface Parcellation

Studies of this region have tended to use inconsistent nomenclature. We mapped four atlases with commonly used sub-regional labels onto our ERC plus TEC population template: (1) manual labels of ERC and TEC based on cortical folding seen in structural MRI (Tward et al., 2017b), (2) automated labels of ERC and parahippocampal gyrus (PHG) based on cortical folding seen in structural MRI (Desikan et al., 2006), (3) labels of posterior medial ERC (pmERC), anterior lateral ERC (alERC), and perirhinal cortex (PRC) based on connectivity patterns seen in functional MRI after manual segmentation of ERC in structural 7T MRI (Maass et al., 2015), and (4) histological labels of intermediate superior ERC, intermediate rostral ERC, intermediate caudal ERC, prorhinal ERC, medial rostral ERC, medial caudal ERC, lateral ERC, sulcal ERC and TEC as identified in an 11T *ex vivo* MRI (Krimer et al., 1997; Miller et al., 2015a).

Manual segmentation of ERC and TEC was performed on an scan with a Type IIa CoS variant of regular depth (1.30 cm). To generate labels from the Desikan-Killiany atlas, FreeSurfer 6.0 was run on the same scan. The functional MRI atlas was provided on a subject with Type IIa CoS variant of regular depth (1.20 cm) (Maass et al., 2015). The *ex vivo* MRI atlas was provided on a subject with a Type IIb CoS variant of shallow depth (0.75 cm). For each atlas, we manually segmented the ERC plus TEC from the structural MRI following the same protocol as for our subjects. In the *ex vivo* case, since the CoS was shallow, the TEC was extended to the lateral bank of the CoS, as seen in histology (Insausti et al., 1998; Ding and Van Hoesen, 2010). We then mapped the atlas labels to the manually-defined ERC plus TEC surface by linear interpolation. Finally, we mapped these surfaces and their labels to the population template surface following the LDDMM framework (Beg et al., 2005). The result was four sets of labels, one from each atlas, on each vertex of the population template surface.

The Desikan-Killiany atlas defined the anterior boundary of ERC at the rostral end of the CoS; this was approximately 6 mm anterior to the boundary defined in our protocol. The functional MRI atlas defined the anterior boundary at the rostral end of the amygdala, which coincided with our protocol's boundary. The anterior boundary on the *ex vivo* MRI atlas was 0.5 mm posterior to our protocol's boundary.

The Desikan-Killiany atlas defined the posterior boundary at the caudal end of the amygdala. This excludes a posterior portion of the ERC that runs lateral to the hippocampal formation (Krimer et al., 1997; Insausti et al., 1998; Ding and Van Hoesen, 2010). The functional MRI atlas defined the posterior boundary as extending to the caudal end of the CoS. Since this atlas was a Type IIa CoS variant, the caudal extent of the CoS coincided with 1.2 mm posterior to the GI, or one 0.6 mm slice anterior to our protocol's boundary. The posterior boundary of the *ex vivo* atlas was 1.0 mm anterior to our protocol's boundary.

**Figure 3:**
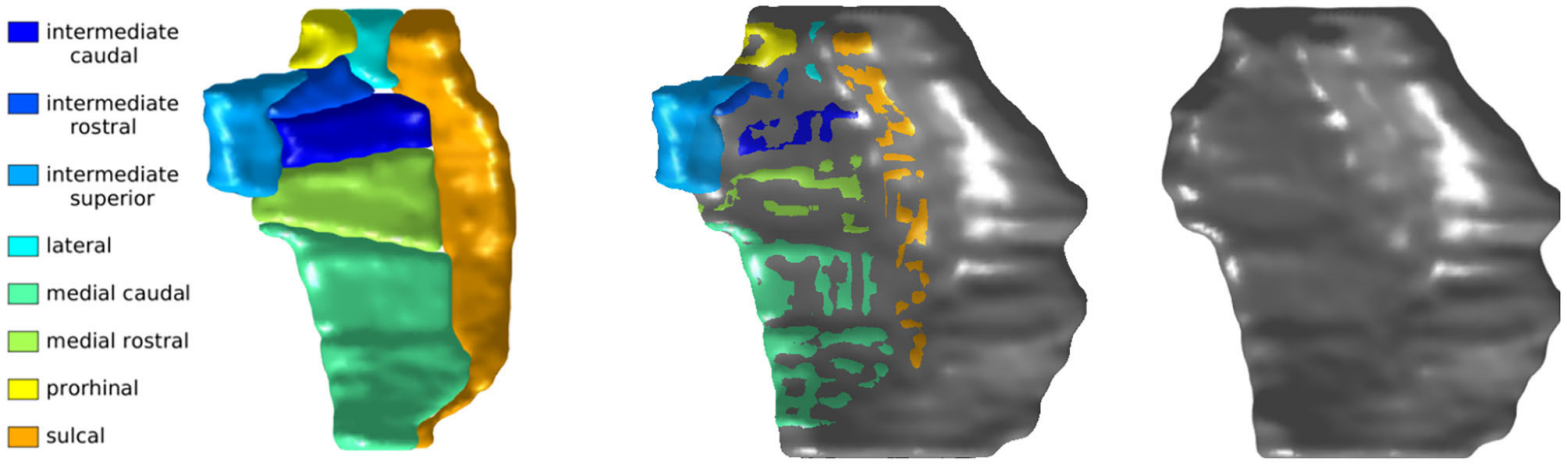
Krimer subregions of ERC labeled on 11T *ex vivo* MRI **(left)**. ERC plus TEC labeled on same MRI following protocol described in section 4.2 **(right)**. Overlay of segmentations **(middle)**. The orientation is as follows: anterior (top), posterior (bottom), medial (left), lateral (right).

The Desikan-Killiany atlas, functional MRI atlas, and our protocol defined the medial boundary at the furthest extent where gray/white boundary was visible. The *ex vivo* atlas included the dorsal medial aspect of the ERC that borders the amygdala, a border that is not always visible on 3T T1 MRI. An overlay of the *ex vivo* atlas ERC partition and our protocol's ERC plus TEC partition highlights this difference as shown in Figure 3.

Finally, the Desikan-Killiany atlas defined the lateral extent of entorhinal cortex as the most lateral extent of the CoS. This boundary definition most closely followed that of a shallow CoS variant (Insausti et al., 1998), often seen in Type IIb CoS that were excluded from this study. The functional MRI atlas delineated the ERC ending at the shoulder of the CoS, a definition that most closely followed the boundary of a deep CoS variant (Insausti et al., 1998). Our protocol also followed the delineation that matches a deep CoS variant. Since the subjects included in this study range from regular CoS depth (1.0–1.5 cm) to deep CoS depth (> 1.5 cm), it is likely that our protocol may include a small portion of the ERC within the TEC label.

### 2.4. Cortical Thickness

To calculate vertex-wise cortical thickness, we followed an established procedure based on LDDMM (Ratnanather et al., 2019). The ERC plus TEC surface was cut into two surfaces: the pial surface and the gray matter-white matter boundary surface. The gray matter-white matter boundary surface was deformed to the pial surface within the LDDMM framework, with an additional imposed constraint that the surface must flow in the direction normal to its evolving surface. Cortical thickness was then estimated as the distance along these trajectories. Average ERC thickness and average TEC thickness were calculated as the mean thickness across vertex labeled ERC and TEC, respected, as mapped from the manual structural MRI atlas.

### 2.5. Group Difference Analysis

We tested where there were differences in shape measures by diagnostic group. The log-linear mixed effects model under the null hypothesis can be written as Equation (1) given a subject *i*, scan *j*, and vertex *k*.

(1) $$\begin{array}{l} {\text{log}(\text{thickness})}_{i,j,k}=a_{k}+b_{k}\;{\text{age}}_{i,j}+c_{k}\;{\text{sex}}_{i}+e_{i,k}+\epsilon_{i,j,k} \end{array}$$

The constants *a*, *b*, *c*, and the variance of 0-mean Gaussians *e* and ϵ are estimated by maximum likelihood. *e* is the subject-specific random effect, while age and sex (a binary indicator variable) are fixed effects.

The log-linear mixed effects model under the alternative hypothesis can be written as Equation (2).

(2) $$\begin{array}{l} {\text{log}(\text{thickness})}_{i,j,k}=a_{k}+b_{k}{\text{age}}_{i,j}\\ \;+\left(a_{k}^{\prime }+b_{k}^{\prime }\left({\text{age}}_{i,j}-{\text{age\_MCIonset}}_{i}\right)\right){\text{ isMCI}}_{i}\\ \;+c\;{\text{sex}}_{i}+e_{i,k}+\epsilon_{i,j,k} \end{array}$$

isMCI is a binary indicator variable for whether a subject belongs to the group that converted from NC to MCI, and age_MCIonset is the age of MCI diagnosis. *a*′ is the mean difference in log thickness at the time of MCI diagnosis for subjects that converted from NC to MCI. *b*′ corresponds to the disease-related rate of change in this group.

We tested whether the model under the alternative hypothesis significantly fit the data better than the model under the null hypothesis by using the likelihood ratio as a test statistic and bootstrap resampling 10,000 samples. The bootstrapped samples were constructed by sampling from whitened residuals under the null hypothesis. We compared the likelihood ratio to the distribution of likelihood ratios of the bootstrapped samples. We corrected for multiple comparisons over the vertices by using the maximum test statistic over all vertices for each set of bootstrapped samples (Nichols and Hayasaka, 2003). We rejected the null hypothesis when the true likelihood ratio was greater than 95% of the bootstrapped likelihood ratios.

### 2.6. Change Point Analysis

We tested when a change in atrophy rate occurred with respect to MCI diagnosis. Details for constructing and testing this change point model are described in another work (Tang et al., 2017). Here we provide a brief summary of the approach.

The log-linear mixed effects model under the null hypothesis can be written as Equation (3) given a subject *i*, scan *j*, and location *k*.

(3) $$\begin{array}{r} {\text{log}(\text{thickness})}_{i,j,k}=a_{k}+b_{k}\;{\text{age}}_{i,j}+c_{k}\;{\text{sex}}_{i}\\ +d_{k}\;{\text{age\_MCIonset}}_{i}+e_{i,k}+\epsilon_{i,j,k} \end{array}$$

The constants *a*, *b*, *c*, *d*, and the variance of 0-mean Gaussians *e* and ϵ are estimated by maximum likelihood. *e* is the subject-specific random effect, while age and sex (a binary indicator variable) are fixed effects. Unlike in the group-wise difference analysis, this model has only two locations in order to reduce computational complexity: one for average ERC thickness, and the other for average TEC thickness.

The model under the alternative hypothesis can be written as Equation (4).

(4) $$\begin{array}{l} {\text{log}(\text{thickness})}_{i,j,k}=a_{k}+b_{k}{\text{age}}_{i,j}\\ \;+b_{k}^{\prime }{\left({\text{age}}_{i,j}-\left({\text{age\_MCIonset}}_{i}+\Delta \right)\right)}^{+}\\ \;+c_{k}{\text{sex}}_{i}+d_{k}{\text{age\_MCIonset}}_{i}+e_{i,k}+\epsilon_{i,j,k} \end{array}$$

Δ is the number of years from a diagnosis of MCI to the change point in atrophy rate, and ${\left({\text{age\_MCIonset}}_{i}+\Delta \right)}^{+}=\text{max}\left({\text{age\_MCIonset}}_{i}+\Delta ,0\right)$ is the number of years past the change point. The constants *a*, *b*, *c*, *d*, and the variance of 0-mean Gaussians *e* and ϵ are estimated by maximum likelihood over a fixed Δ with yearly increments of Δ between −50 and 50 years. The best candidate Δ is calculated from the posterior mean.

For stable NC subjects, we estimated MCI diagnosis from a conditional probability distribution where the age of onset was constrained to be after the last diagnostic evaluation, and drawn from a Gaussian distribution with mean age of μ_1_ = 93 years and standard deviation of σ_1_ = 14.5 years. This distribution of MCI diagnosis was estimated (Tang et al., 2017) using a set of 1, 000 subjects enrolled with normal cognition and a family history of Alzheimer's disease.

Since the subjects in this study were selected to meet diagnostic group criteria and do not represent a random sample over the progression of the disease, we re-weighted the likelihood function by the distribution of stable NC and NC to MCI converters expected if the subjects were selected blind to diagnostic group. The subjects in the ADNI database were enrolled to meet a set number of subjects per diagnostic group, and as such, cannot be used to estimate this distribution. Instead, we examined the BIOCARD database, where subjects were enrolled cognitively normal and followed for up to 22 years at time we examined this database (biocard-se.org). This distribution was calculated from a subset of subjects over 65 years of age at their most recent follow-up visit. Specifically, the proportion was 184/260 stable NC and 44/260 NC to MCI converters.

We tested whether the model under the alternative hypothesis significantly fit the data better than the model under the null hypothesis using the likelihood ratio as a test statistic and bootstrap resampling 1,000 samples. The bootstrapped samples are constructed by sampling from whitened residuals under the null hypothesis, with imputed values for age_MCIonset for stable NC subjects. We compared the likelihood ratio to the distribution of likelihood ratios of the bootstrapped samples and rejected the null hypothesis when the likelihood ratio was greater than 95% of the bootstrapped likelihood ratios. We used the maximum test statistic over pairs of ERC and TEC statistics to control family-wise error rate (Nichols and Hayasaka, 2003).

In the case where the null hypothesis was rejected, we then determined the confidence interval for Δ, *b* and *b*′ by bootstrap resampling under the alternative hypothesis 1,000 times. The bootstrapped samples were constructed by sampling from whitened residuals under the alternative hypothesis, with imputed values for age_MCIonset for NC subjects. In the case where the null hypothesis was rejected for both ERC and TEC measures, we then calculated the probability that the change point for TEC occurred before the change point for ERC based on the change point Δ of each pair of bootstrapped samples under the alternative hypothesis. This one-sided test was selected based on histological evidence that changes occurred in the TEC before the ERC (Braak et al., 2006).

## 3. Results

### 3.1. Surface Parcellation

**Figure 4:**
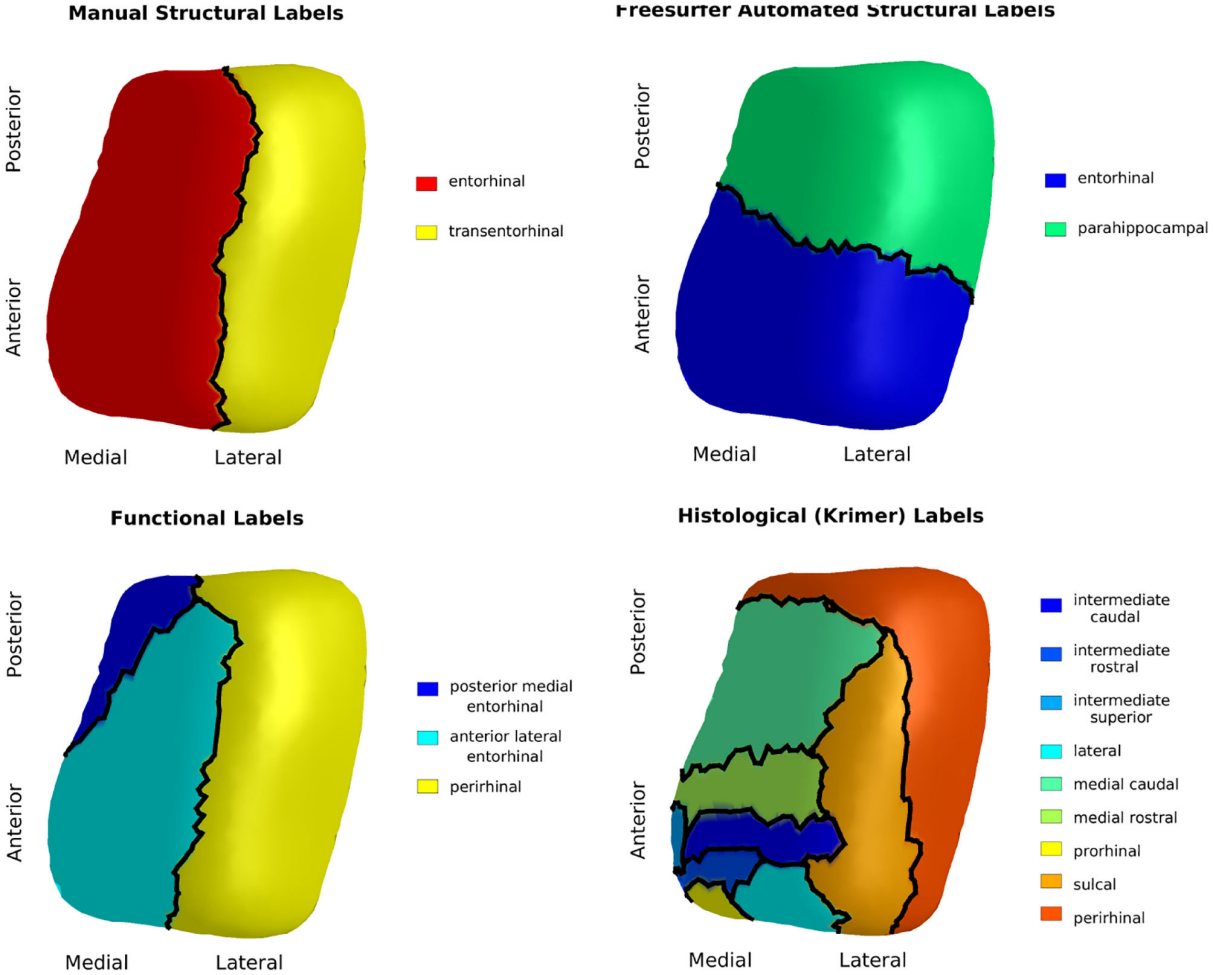
Manual structural labels **(top left)**, automated structural labels **(top right)**, functional labels **(bottom left)**, histological labels **(bottom right)** mapped onto the population template.

The results of the four atlas mappings onto the population template surface are shown in Figure 4. Comparison of the atlases highlight inconsistencies introduced by varying nomenclature and CoS variant. The automated labels based on cortical folding used by Desikan-Killany defined the ERC extending into the CoS, a definition that matches a shallow CoS variant. The atlas also excluded the posterior extent of the ERC. In contrast, the functional MRI atlas defined the perirhinal cortex (PRC), an area that includes the TEC, and separated this structure from the ERC using a definition that matches a deep CoS variant. Histologically-defined subregions of the ERC show yet another popular parcellation of this region on a regular depth (1–1.5 cm) CoS variant. Note how in this definition, the sulcal ERC extends into the shoulder of the CoS. In this study, average ERC and average TEC metrics were calculated based on the labels shown in the manual structural labels (top left).

### 3.2. Cortical Thickness

**Figure 5:**
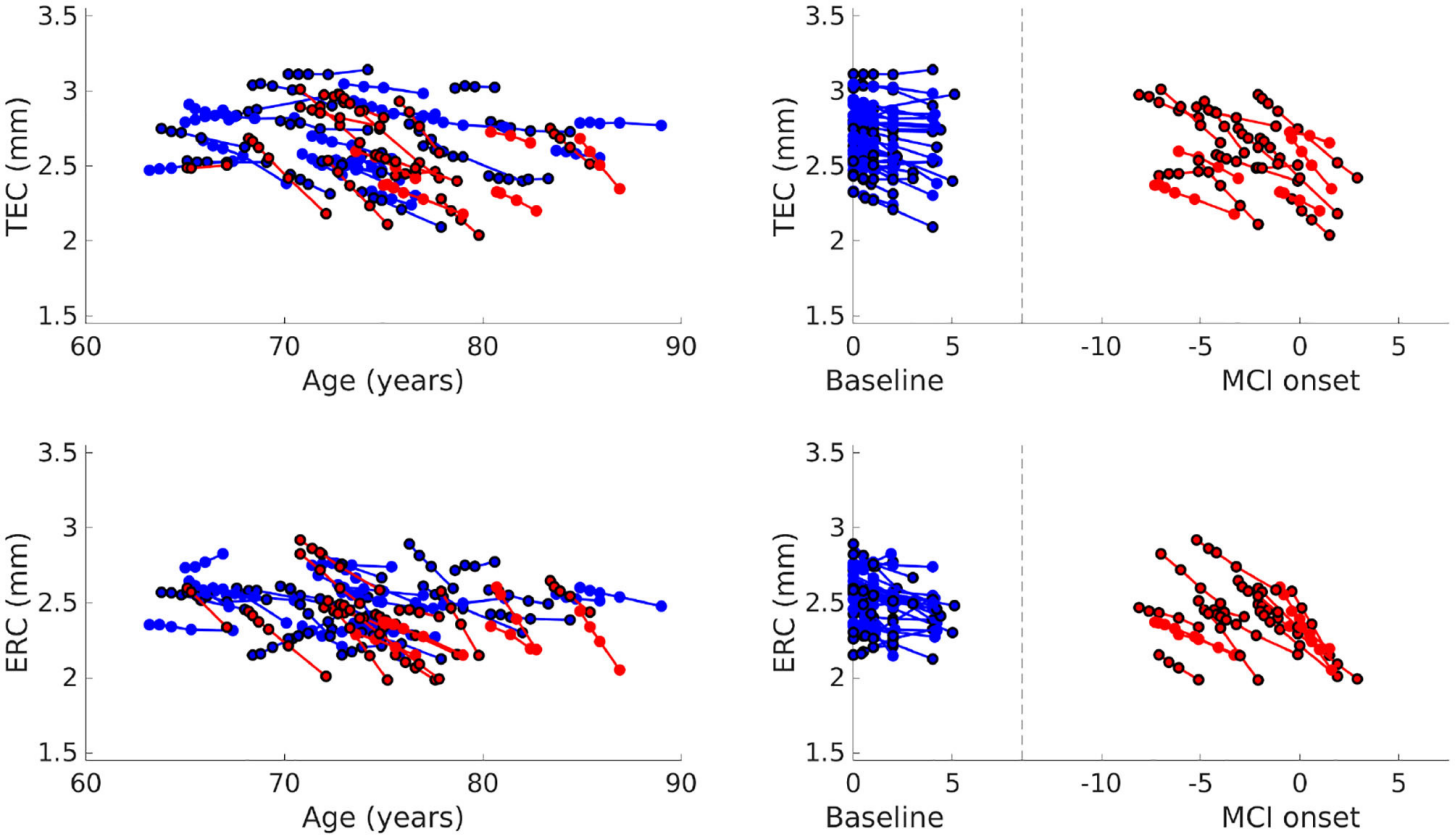
Average TEC thickness **(top)** and ERC thickness **(bottom)** plotted over age **(left)**. Average TEC thickness **(top)** and ERC thickness **(bottom)** plotted with respect to time from baseline scan for NC, and with respect to time from MCI onset for NC to MCI converters **(right)**. Each line corresponds to a subject, the color to a diagnostic group (stable NC, NC to MCI), and the marker border to sex (no border is male, bordered is female).

Average cortical thickness of TEC and average cortical thickness of ERC are plotted in Figure 5. The TEC is slightly thicker than the ERC, which is in agreement with previous work (Kulason et al., 2019). In both regions, organization by MCI diagnosis date show cortical thickness measures decrease with progression of the disease, and that the rate of cortical thinning is noticeably steeper in NC to MCI converters than in stable NC subjects.

### 3.3. Diagnostic Group Difference Analysis

**Figure 6:**
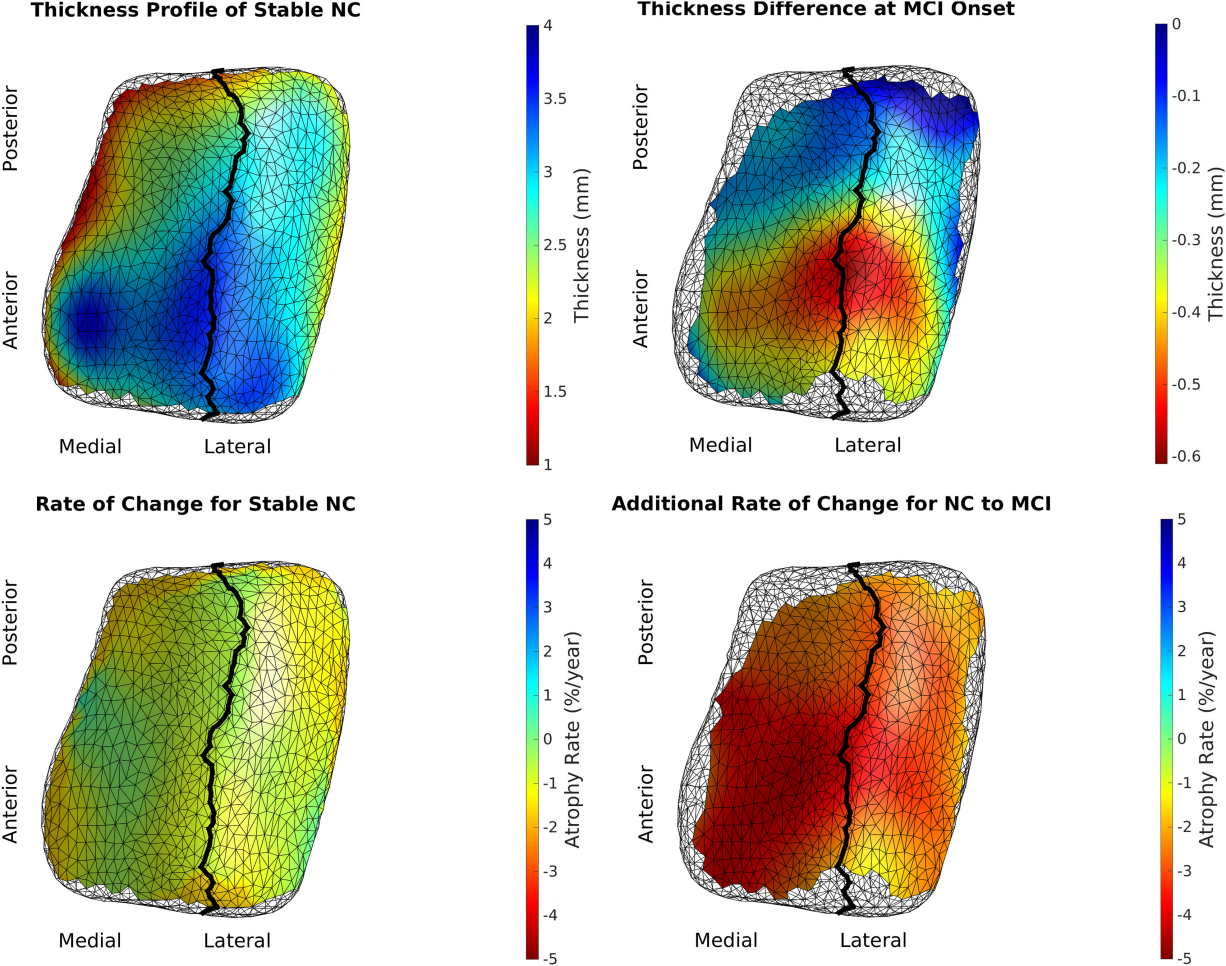
Top left is the model of a 65-year-old, male, stable NC. Bottom left is the age-related atrophy rate. Top right is the difference in thickness at time of MCI diagnosis in NC to MCI converters. Bottom right is the additional atrophy rate for NC to MCI converters.

We rejected the null hypothesis with global *p* < 0.0001 and concluded that there was a difference in cortical thickness between diagnostic groups. Figure 6 shows the difference in atrophy and atrophy rate across all vertices. A summary of average and maximum atrophy/atrophy rates is shown in Table 3. As the data demonstrates, at the time of MCI diagnosis, individuals who had progressed to a diagnosis of MCI were as much as 0.58 mm thinner in the ERC and 0.60 mm thinner in the TEC. The additional atrophy rate in the participants who progressed from NC to MCI was 2.96% per year in the ERC on average and 2.43% per year in the TEC on average. This is a notable increase from age-related atrophy which was, on average, 0.68% per year in the TEC and 0.66% per year in the ERC. In other words, the average total atrophy rate in NC to MCI converters was 5.35 times greater in the ERC and 4.68 times greater in the TEC compared to stable NC subjects.

**Table 3:** Summary of group-wise difference analysis by region.

	**ERC average**	**ERC max**	**TEC average**	**TEC max**
Atrophy at				
MCI diagnosis	0.25 mm (8.46%)	0.58 mm (16.54%)	0.23 mm (7.63%)	0.60 mm (17.34%)
MCI-related				
Atrophy rate	2.96 %/year	4.23 %/year	2.43 %/year	4.11 %/year
age-related				
Atrophy rate	0.68 %/year	2.08 %/year	0.66 %/year	1.61 %/year

### 3.4. Change Point Analysis

We rejected the null hypothesis and concluded that there was a change point 9.02 years before MCI onset for the ERC (*p* < 0.001) and 10.69 years before MCI diagnosis for the TEC (*p* < 0.001). Prior to the change point, the atrophy rate was 0.35%/year for the ERC and 0.34%/year for the TEC. After the change point, the additional atrophy was 3.75%/year for the ERC and 2.58%/year for the TEC. The 95% confidence interval for the parameters of interest are shown in Table 4. The ERC change point in thickness occurred at or before the TEC change point in 3.75% of bootstrapped samples. We concluded that the TEC change point preceded the ERC change point.

**Table 4:** 95% confidence interval (min, max).

	**Change point (years before MCI)**	**Age-related rate (%/year atrophy)**	**Disease-related rate (%/year atrophy)**
ERC thickness	(7.63, 11.31)	(0.07, 0.65)	(3.03, 4.41)
TEC thickness	(8.92, 13.80)	(0.10, 0.56)	(2.11, 3.08)

*Change point, age-related rate, and disease-related rate correspond to the variables −Δ, −b, and −b′, respectively. The disease-related rate is the additional rate seen post change point*.

## 4. Discussion

The first major finding of this study is that anterior regions of the ERC and TEC were more than half a millimeter thinner (up to 17% thinner) in NC to MCI converters at the time of MCI diagnosis, and that disease-related atrophy was roughly 3% per year. The evidence suggests that disease-related atrophy begins prior to an MCI diagnosis in the anterior lateral region of ERC and anterior region of TEC. This is in line with our previous study that examined subjects after an MCI diagnosis, where we found disease-related thickness atrophy was 5% atrophy per year in the TEC and that MCI subjects were on average 23% thinner than NC (Kulason et al., 2019). The 3% atrophy per year in NC to MCI converters vs. 5% atrophy per year in subjects after MCI diagnosis may suggest that the atrophy rate increases with progression of the disease from the preclinical to clinical stage.

The second major finding of this study is that there was a change in the rate of ERC thickness atrophy 8–11 years prior to MCI diagnosis, and a change in the rate of TEC thickness atrophy 9–14 years before the diagnosis of MCI. The order of the change points, TEC before ERC, is consistent with histological report of neurofibrillary tau accumulation in this region (Braak et al., 2006). The time of change point is consistent with a previous study, which found changes in surface area of the FreeSurfer-defined ERC 8–10 years prior to symptom onset (Younes et al., 2014).

These findings emphasize the discrepancies in nomenclature pertaining to the TEC and ERC. We showed how the Desikan-Killiany atlas defined ERC beginning anterior to our protocol and ending anterior to our protocol. Our protocol closely matched the anterior and posterior boundaries defined in the function MRI atlas and *ex vivo* atlases. The Desikan-Killiany ERC extended laterally into the CoS, which is accurate for shallow CoS common variant in Type IIb CoS, and not as accurate for the regular and deep CoS variants that were included in this study. The *ex vivo* atlas, a subject with Type IIa CoS variant of regular depth, marked the sulcal ERC lateral boundary shortly past the shoulder of the CoS, which is consistent with cytoarchitectonically-defined ERC in previous studies (Krimer et al., 1997; Insausti et al., 1998; Ding and Van Hoesen, 2010). The functional MRI atlas and Desikan-Killiany atlas chose not to define the TEC separate from perirhinal cortex and fusiform gyrus, respectively. The 4 surface parcellations mapped to the same coordinate system highlight the need for a standardized nomenclature of this region, much like the work being done to standardize sub-regional boundaries of the hippocampus (Adler et al., 2018; Olsen et al., 2019).

There are a number of strengths to this study. The subjects were carefully selected to (1) follow a strict set of inclusion criteria for diagnostic grouping, and (2) exclude subjects with a discontinuous CoS within the region of interest. This was done to reduce confounding factors introduced by other medical conditions and natural variability in cortical folding. In addition, scans were manually segmented to avoid errors introduced by automated segmentation methods. Finally, the results were put in the context of several atlases for broader interpretation of findings.

There are also a few limitations to this study, the first being a relatively small sample size. Accurate segmentations, which have been performed manually for this study, are extremely time consuming. The ERC is particularly difficult to segment automatically due to its proximity with the meninges and oculomotor nerve. These neighboring structures are a similar intensity to gray matter voxels in T1 scans. Some recent work has been done to address this issue using automatic parcellation (Xie et al., 2017); a future direction is to determine whether this type of automated approach to segmentation affects ERC and TEC metrics produced in this analysis.

Another limitation of this study is that the distribution of samples is biased by diagnostic grouping. It is difficult to estimate the true distribution of diagnostic groups because the ADNI protocol selected subjects based on diagnosis and the follow-up time varies. This is mitigated by the use of distribution estimates calculated from the BIOCARD database.

In the future, this study can be extended to include shallow, discontinuous CoS variants for detecting Alzheimer's-related changes. It is of interest to develop metrics of disease progression that are robust to this natural variation in folding. Autopsy studies have shown that subjects with a shallow, discontinuous CoS have a TEC that begins at the deepest extent of the CoS and extends out laterally, whereas deep, continuous CoS have a TEC that begins at the shoulder of the CoS and extends only to the deepest extent of the CoS (Insausti et al., 1998; Ding and Van Hoesen, 2010). Therefore, a multi-atlas approach with CoS variant-specific atlases may be desirable to delineate the ERC and TEC accurately for a full population of subjects.

Finally, given the localization of tau to CA1 after initial deposits along the boundary of TEC and ERC (Braak and Braak, 1991), it is of interest to extend thickness analysis to this region. Unfortunately, the CA1 subfield cannot be segmented separately from the hippocampal formation in 3T T1 MRI. Previous volumetric analysis of the hippocampus has shown 9% atrophy in MCI subjects, compared to 27% volumetric atrophy of ERC plus TEC in MCI subjects (Kulason et al., 2019). Previous change point analysis of surface expansion/contraction metrics have shown a hippocampal change point 2–4 years prior to symptom onset, compared to 8–10 years prior to symptom onset for a Desikan-Killiany defined ERC (Younes et al., 2014). As more high resolution T2 MRI data become available, such as with data being collected in the more recent ADNI 3 protocol, it will be of interest to extend this thickness analysis to the CA1 subfield.

This study provides strong evidence that TEC and ERC thickness is a sensitive measure to progression to the symptomatic phase of Alzheimer's disease and that disease-related atrophy begins to occur at least 9 years prior to a clinical diagnosis of MCI.

## Data Availability Statement

The data analyzed in this study is subject to the following licenses/restrictions: Individuals need to register with ADNI and agree to the conditions in the “ADNI Data Use Agreement” and undergo limited screening by the DPC before accessing data. Requests to access these datasets should be directed to http://adni.loni.usc.edu/data-samples/access-data/.

## Ethics Statement

The studies involving human participants were reviewed and approved by Good Clinical Practice guidelines, the Declaration of Helsinki, and the US 21 CFR Part 50-Protection of Human Subjects, and Part 56-Institutional Review Boards (IRB). The patients/participants provided their written informed consent to participate in this study.

## Author Contributions

SK and EX processed the data. SK performed the analysis, drafted the manuscript, and designed the figures. DT, MA, LY, and MM contributed to the design and implementation of the research and to the analysis of the results. All authors contributed to the editing of the manuscript.

## Conflict of Interest

The authors declare that the research was conducted in the absence of any commercial or financial relationships that could be construed as a potential conflict of interest.
